# Case Report: CD19 CAR-T therapy induces dual remission in AML-M2b patient with CNS-PTLD and relapse

**DOI:** 10.3389/fimmu.2025.1672392

**Published:** 2025-11-05

**Authors:** Yu Zhang, Haibo Zhu, Xia Xiao, Mingfeng Zhao

**Affiliations:** Department of Hematology, Tianjin First Central Hospital, Tianjin, China

**Keywords:** CD19 CAR-T, AML-M2b, extramedullary relapse, post-transplant lymphoproliferative disorder, case report

## Abstract

**Background:**

Acute myeloid leukemia (AML)-M2b with t(8;21)(q22;q22)/RUNX1::RUNX1T1 (AML1-ETO) is associated with a high risk of relapse after allogeneic hematopoietic stem cell transplantation (allo-HSCT). Post-transplant lymphoproliferative disorder (PTLD), particularly involving the central nervous system (CNS), confers a poor prognosis. Although CD19 chimeric antigen receptor T-cell (CAR-T) therapy is established in B-cell malignancies, its application in acute myeloid leukemia (AML) or CNS-PTLD has rarely been reported.

**Case:**

A 24-year-old male with AML-M2b showed persistent RUNX1::RUNX1T1 (AML1-ETO) positivity after allo-HSCT. He developed an extramedullary relapse (presacral mass) at 7 months, followed by CNS-PTLD with limb palsy at 9 months post-HSCT. The disease subsequently progressed to bone marrow relapse (RUNX1::RUNX1T1 94.42%, MRD >5%).

**Intervention:**

Given the co-expression of CD19 on both the AML and PTLD cells, the patient was treated with donor-derived CD19 CAR-T cells. He experienced manageable grade 1 cytokine release syndrome (CRS) and grade 3 Immune Effector Cell-Associated Neurotoxicity Syndrome (ICANS).

**Outcomes:**

The patient achieved a complete response (CR) with negative MRD, disappearance of the fusion gene, reduction of PTLD and extramedullary lesions, and recovery of limb strength.

**Conclusion:**

This case demonstrates the efficacy and feasibility of CD19 CAR-T therapy for concomitant post-transplant AML-M2b relapse and CNS-PTLD, leveraging their shared CD19 expression. It provides clinical evidence that targeting a shared antigen with a single CAR-T product can effectively treat heterogeneous malignancies, offering a promising new strategy for such complex cases.

## Introduction

Acute myeloid leukemia (AML)-M2b, characterized by t(8;21)(q22;q22) translocation (1), is driven by the RUNX1::RUNX1T1 (AML1-ETO) fusion oncoprotein. Allogeneic hematopoietic stem cell transplantation (allo-HSCT) is a curative option for AML-M2b, but post-transplant relapse remains a major challenge with poor prognosis. Notably, emerging research has revealed that a subset of AML patients, particularly those with t(8;21)-positive AML-M2b, may exhibit aberrant expression of CD19, and early case reports indicated that CD19-targeted chimeric antigen receptor T-cell (CAR-T) therapy could be effective (2–4).

Post-transplant lymphoproliferative disorder (PTLD) is a severe complication following allo-HSCT, with 60%-80% associated with Epstein-Barr virus (EBV) reactivation (5, 6). Central nervous system involvement (CNS-PTLD) occurs in 5%-20% of PTLD cases (7) and carries a dismal prognosis. This is largely attributed to the blood-brain barrier (BBB) and the immune-privileged status of the CNS. Conventional therapies, including immunosuppressant dose reduction, antiviral therapy, and chemotherapy, show limited efficacy, highlighting the need for novel approaches. CAR-T cells may cross the BBB, while clinical data for CNS-PTLD remain scarce.

This report describes the first case of AML-M2b with post-HSCT relapse and concurrent CNS-PTLD that achieved dual remission following CD19 CAR-T therapy. This successful outcome proposes a novel therapeutic paradigm for such complex cases and underscores the potential of CAR-T therapy in targeting heterogeneous malignancies.

## Case presentation

A 24-year-old male patient was initially diagnosed with AML-M2b at an external hospital in April 2023. Cytogenetic analysis revealed a chromosomal karyotype of 45, X,−Y, t(8;21)(q22;q22), and transcripts of the RUNX1::RUNX1T1 (AML1-ETO) fusion gene were detected. Genetic testing identified several mutations, including WT1 (11.29%), EVI1 (0.2%), and potential germline mutations in CSF3R and CREBBP (P.Ser602Arg). The patient underwent induction chemotherapy with homoharringtonine (HHT), azacitidine (AZA), and a BCL-2 inhibitor (bcl2i). Then, he received consolidation therapy, including a CAG regimen, AZA plus arsenic trioxide (AAG), and regimens containing intermediate - dose cytarabine (Ara-c). Although minimal residual disease (MRD) remained low (0.01%), the RUNX1::RUNX1T1 (AML1-ETO) fusion transcript remained positive, indicating persistent disease activity.

Consequently, the patient underwent allogeneic hematopoietic stem cell transplantation (allo-HSCT) in February 2024, using a modified BUCY conditioning regimen. He received unrelated cord blood stem cells (male donor, 6/10 HLA match, A+ blood type for AB+ recipient) and peripheral stem cells from his father (5/10 HLA match, A+ blood type for AB+ recipient). The total infused mononuclear cell (MNC) count was 9.37×10^8^cells/kg, with CD34+ cell count of 0.84×10^6^ cells/kg. Post-transplant remission was short-lived, with the RUNX1::RUNX1T1 (AML1-ETO) fusion gene becoming detectable again on day 42 and increasing to a level of 0.35%. Decitabine and donor lymphocyte infusion (DLI) briefly reduced the fusion gene levels but induced intestinal graft-versus-host disease (GVHD).

**Figure 1:**
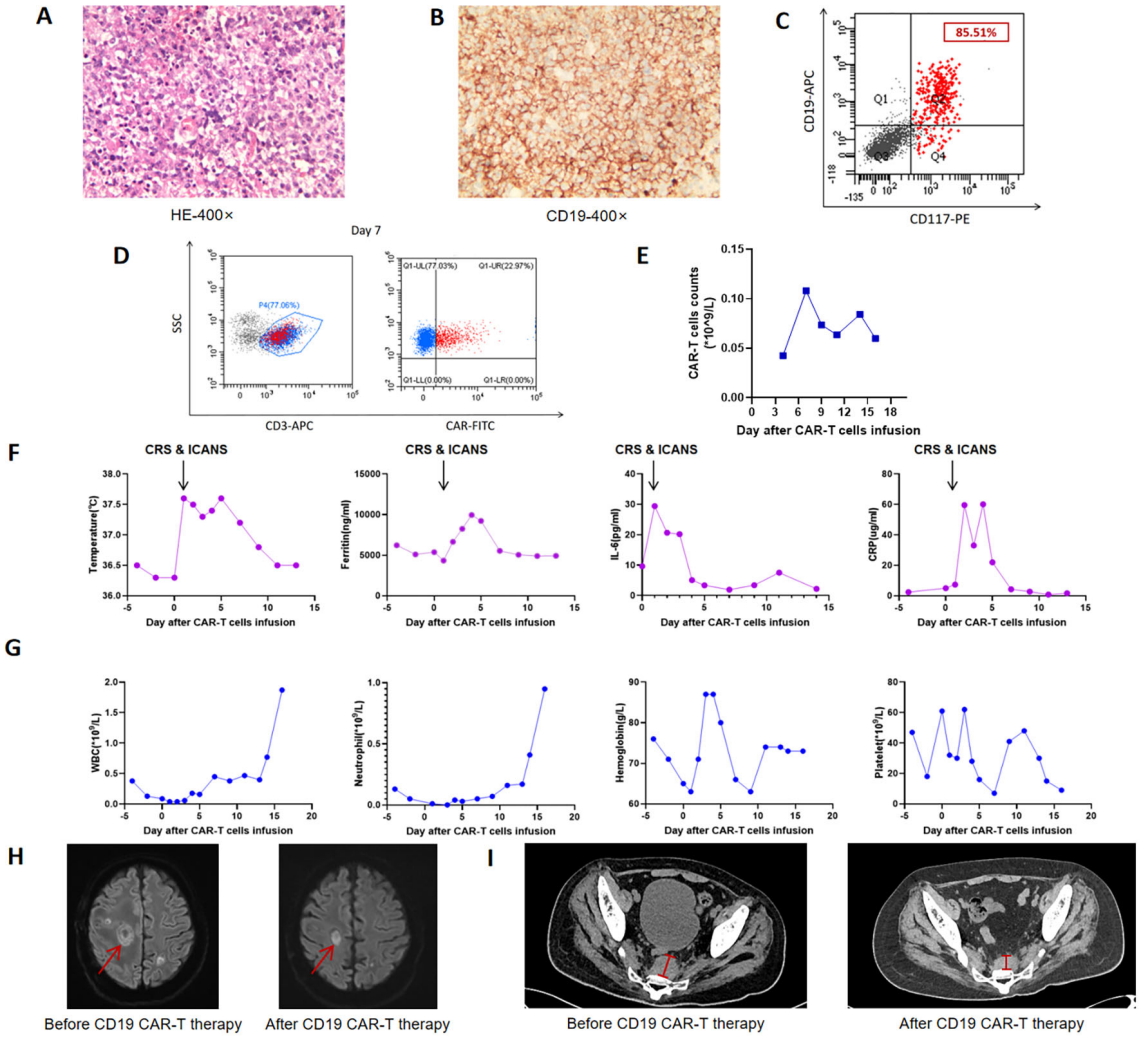
Clinical data during CAR-T cell therapy. **(A)** Hematoxylin and eosin (H&E) staining of intracranial tissues; **(B)** Immunohistochemistry of intracranial tissues showing high expression of CD19 (100%); **(C)** Flow cytometric analysis detecting CD19 expression on 85.51% of AML blasts; **(D)** Flow Cytometry analysis illustrating the expansion of CAR-T cells within the population of peripheral CD3-positive cells on Day +7; **(E)** Kinetics of CAR-T cell expansion in peripheral blood; **(F)** Changes in body temperature, ferritin, interleukin-6 (IL-6), and C-reactive protein (CRP) levels during therapy; **(G)** Changes in peripheral blood cell counts, including white blood cells (WBC), neutrophils, hemoglobin, and platelets (PLT); **(H)** Comparative head magnetic resonance imaging (MRI) scans before and after CAR-T treatment; **(I)** Comparative imaging of recurrent presacral soft tissue lesions before and after CAR-T treatment. CRS, Cytokine release syndrome; ICANS, Immunoeffector cell-associated neurotoxicity syndrome.

Unfortunately, 7 months post-HSCT, a presacral soft tissue mass suggested extramedullary relapse. At 9 months, the patient presented with dysarthria, seizures, and complete loss of muscle strength (grade 0/5) in the left limb. Cerebrospinal fluid (CSF) analysis revealed elevated protein levels, and next-generation sequencing (NGS) of the CSF detected 1335 copies of EBV sequence. Head contrast-enhanced MRI revealed multiple lesions. Surgical pathology confirmed CNS-PTLD (EBV-positive diffuse large B-cell lymphoma), with tumor cells positive for CD19 (100%, **Figures 1A, B**). The brain tissue NGS test indicated an EBV copy number of 6699, which was at its peak. Subsequent treatment with rituximab and methotrexate resulted in the CSF EBV copy number falling below the detection limit. The patient has experienced slight improvement in left limb movement and sensation, but he still has occasional episodes of brief loss of consciousness.

**Figure 2:**
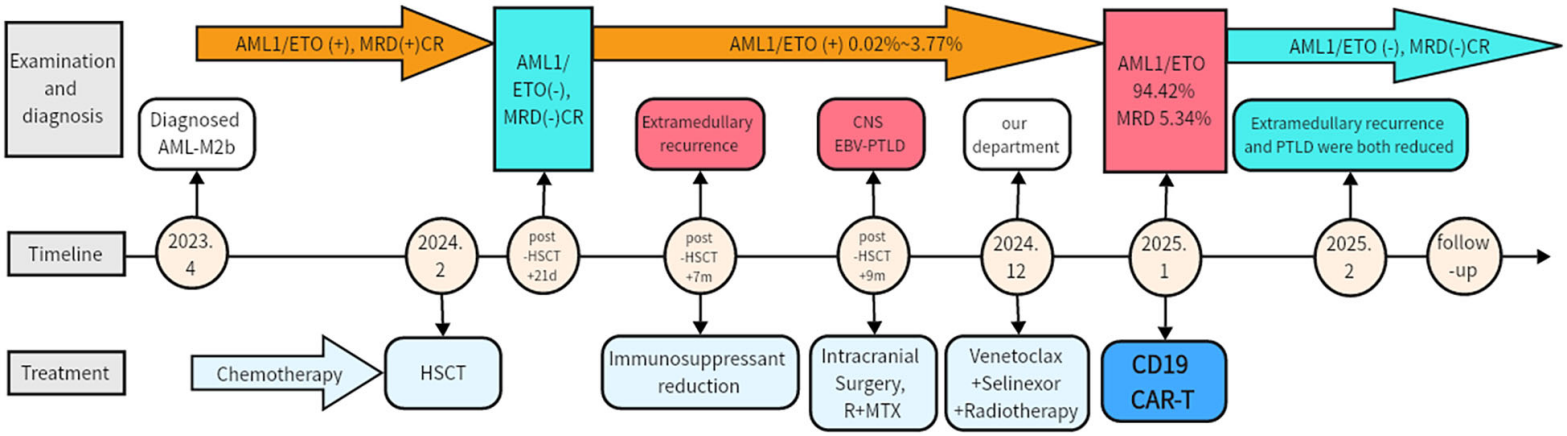
Clinical course and treatment timeline. MRD, Minimal residual disease; CR, complete response; HSCT, Hematopoietic stem cell transplantation; EBV-PTLD, Epstein-Barr virus post-transplant lymphoproliferative disorder; CNS, Central nervous system; R, Rituximab; MTX, Methotrexate.

Subsequently, the patient was admitted to our hospital in December 2024. Following radiotherapy of the brain, the muscle strength of the left limb partly improved. Reevaluation with dynamic contrast-enhanced head MRI showed partial reduction in the size of multiple abnormal signals and decreased local enhancement. But later, bone marrow MRD showed 5.34% blasts with 85.51% CD19 expression (**Figure 1C**), and RUNX1::RUNX1T1 (AML1-ETO) surged to 94.42%. Given the co-expression of CD19 on both PTLD and AML cells, donor-derived CD19 CAR-T therapy was administered at a dose of 1×10^6^ and 1.9×10^6^ cells/kg after conditioning with fludarabine (30 mg/m² per day) and cyclophosphamide (300 mg/m² per day). The peak of CAR-T expansion (22.97% in peripheral blood) occurred at day 7 (**Figures 1D, E**). Additionally, the trends of temperature, inflammatory factors, and blood routine parameters during CAR-T treatment are shown in **Figures 1F, G**. Grade 1 cytokine release syndrome (CRS) and grade 3 Immunoeffector cell-associated neurotoxicity syndrome (ICANS) occurred on the first day after CAR-T infusion. Dexamethasone was administered for immune modulation, while valproic acid and intermittent phenobarbital controlled seizure activity. Additionally, mannitol was used to enhance brain dehydration. The patient had no further seizures 2 days after initiating this intervention, with gradual resolution of neurotoxic symptoms thereafter. No obvious acute or chronic GVHD signs were observed. Follow-up imaging showed significant reduction in brain and presacral lesions (**Figures 1H, I**). Currently, the muscle strength of the left upper limb has recovered to grade 4, greatly improving the quality of life. The patient is currently under follow-up to observe the durability of the CD19 CAR-T treatment (current duration 5 months). The entire treatment process and assessment of remission are comprehensively illustrated in **Figure 2** and **Supplementary Figure 1**.

## Discussion

Acute myelogenous leukemia (AML)-M2b, characterized by the (8;21)(q22;q22) translocation leading to the RUNX1::RUNX1T1 (AML1-ETO) fusion gene, exhibits unique biological features that predispose to extramedullary relapse and treatment resistance. The RUNX1::RUNX1T1 (AML1-ETO) oncoprotein disrupts hematopoietic differentiation, promoting stem cell self-renewal and myeloid blast expansion (8). Notably, 78%-81% of AML1-ETO-positive cases aberrantly express CD19 (9–12), a B-cell lineage marker. The aberrant CD19 expression in t(8;21) acute myeloid leukemia is primarily driven by the AML1/ETO fusion protein, which induces expression of the B-cell transcription factor PAX5. PAX5 in turn binds to the CD19 promoter and enhancer to activate its transcription (13, 14). This expression is not merely a phenotypic aberration but has clinical relevance, as evidenced by higher complete remission rates with CD19 CAR-T therapy in CD19-positive AML-M2b patients. Our case underscores that CD19 positivity in AML-M2b (85.51% of blasts) can be leveraged for targeted therapy. Liu et al. reported a complete remission (CR) rate of 57.1% in 7 AML patients (2). Similarly, a Phase 2 trial demonstrated a 66.7% CR rate in relapsed/refractory (r/r) t(8;21) AML patients, with a median duration response of 8.5 months (3). Similarly, a prospective phase II clinical trial (NCT03896854) achieved 100% CR in 10 patients, though median leukemia-free survival was only 3.8 months (4), highlighting the need for consolidation therapy.

Post-transplant lymphoproliferative disease (PTLD) is a life-threatening complication of allo-HSCT. EBV-driven B-cell type PTLD accounts for over 90% of all cases. Central nervous system involvement of PTLD (CNS-PTLD) accounts for only 5%–20% of PTLD cases (7). It has a median survival of less than 6 months (5), largely due to the immune-privileged status of the CNS and the blood-brain barrier (BBB). Conventional therapies, such as immunosuppressant dose reduction, antiviral therapy, chemotherapy, rituximab administration, and local radiotherapy or surgical intervention, have limited efficacy. CD19 CAR-T therapy showed promise in solid organ transplantation (SOT)-associated PTLD, with an 82.4% overall objective response rate (ORR) and 58.5% CR rate in small series (15–18). Notably, there is limited literature on allo-HSCT-related PTLD, particularly CNS-PTLD. Recent investigations have demonstrated the feasibility and safety of CAR-T cell therapy, including CD19 CAR-T based approaches, in patients with CNS lymphoma (19–21). The ability of CAR-T cells to cross the BBB and be detected in the cerebrospinal fluid offers a potential therapeutic strategy for CNS-PTLD. In our case, donor-derived CD19 CAR-T cells successfully crossed the BBB, as evidenced by lesion regression and clinical recovery. Notably, CNS-PTLD tumor cells expressed CD19 uniformly, a rarity in solid tumors. This homogeneity may explain the robust response.

The patient has maintained complete remission for 5 months post-CD19 CAR-T infusion. Key indicators include undetectable RUNX1::RUNX1T1 (AML1-ETO) fusion gene, negative bone marrow MRD, and reduced CNS/presacral lesions on follow-up imaging. The primary limitation of this report is the short duration of follow-up. Late relapse cannot be fully excluded. The main mechanisms of escape from CD19 CAR-T therapy in heterogeneous tumors include the outgrowth of pre-existing or newly evolved CD19-negative subclones and impaired CAR-T cell cytotoxicity due to T-cell exhaustion or suppression within the tumor microenvironment. Furthermore, targeting a single antigen inherently amplifies the risk of relapse, as it lacks redundant targeting mechanisms to counter tumor heterogeneity and selectively favors the expansion of antigen-loss variants. Longer-term surveillance will be critical to validate the durability of remission, and we plan to update this clinical outcome in future follow-up reports.

In this study, we have reported a rare case of targeting dual malignancies with CD19 CAR-T therapy via CD19 co-expression on CNS-PTLD and AML-M2b. The treatment resulted in significant efficacy, achieving dual remissions with manageable side effects. Overall, the strategy of targeting shared antigens across tumor types may revolutionize combinatorial immunotherapy for complex post-transplant syndromes.

## Data Availability

The raw data supporting the conclusions of this article will be made available by the authors, without undue reservation.

## Supplementary material

Supplementary Figure 1 The timelines of the radiotherapy and CAR-T therapy.
