# Supplementary figures and images for: Case Report: CD19 CAR-T therapy induces dual remission in AML-M2b patient with CNS-PTLD and relapse
Source: Front Immunol. 2025 Nov 5;16:1672392. doi: 10.3389/fimmu.2025.1672392 (PMC12627000; doi:10.3389/fimmu.2025.1672392)

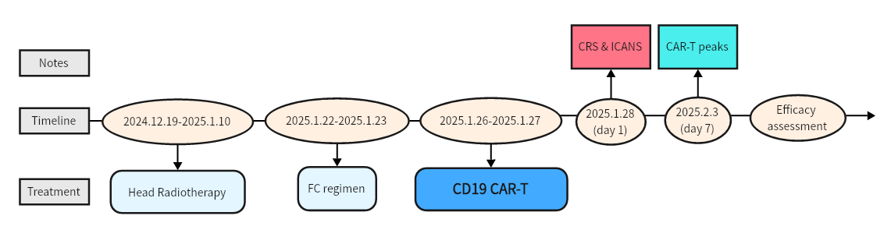

Supplement: Supplementary Figure 1 — The timelines of the radiotherapy and CAR-T therapy. [file Image1.tif]
